# A Physician’s Problems in Psychiatry

**Published:** 1881-03

**Authors:** 


### III. A PHYSICIAN'S PROBLEMS IN PSYCHIATRY.

Dr. C. H. Hughes, of St. Louis: Psychiatry is preëminently a practical subject. Comparatively few general practitioners have had adequate opportunity afforded them of practically studying this disease. The protean phase of mental alienation is mainly to be seen under the present methods of teaching the insane in the hospitals. This fact adds perplexity to the mode of properly treating and disposing of insane persons when we encounter them in general practice. When in the course of physical disease mental alienation, either alone or jointly with another organ, supervenes, the absence of that clinical experience which comes from daily intercourse with the insane and daily observation of the disease, and which usually enables the general practicioner to make a prompt and satisfactory decision in diseases he is accustomed more often to encounter, is likely to occasion every kind of hesitation and indecision. This often results in too hasty and indiscriminate consignment of patients to State hospitals. How can a physician determine when a given case can be safely treated at home? To determine this properly the physician must ascertain whether the patient is homicidal, suicidal, violent or destructive in any way to the person or property of others, or to himself, beyond the likelihood of ordinary home vigilance to prevent or circumvent, and he must also determine whether the patient is in imminent danger of becoming so. Is the patient so indecent in his habits, conduct or language or ordinary proprieties of life as to render it unfit for him to remain long at home? What antipathies has he formed? Is there danger to wife, husband or children, or is their presence detrimental to his mental welfare? Can he be treated safely by medicine alone? Was the insanity caused or is it aggravated by any circumstances surrounding him? What is the pecuniary condition of the family? And many other practical questions are to be solved.

(Dr. Hughes next pointed out at length the importance of this subject and the necessity of great wisdom in making discriminations between those proper to be sent to asylums and those not proper to be sent to asylums.)

Sufferers are sometimes sent to the asylum too early and sometimes too late. There is great need of a more general knowledge of psychiatry out of the asylums. In no class of affections is it so imperatively necessary to encourage the importance of early and prompt treatment, as in diseases of the brain affecting the mind. Among the cases which I have seen too soon or needlessly sent to the asylam are, first, puerperal insanity, during the first six weeks, in consequence of parturient shocks, exhaustive discharges and inadequate nutrition. (And let me say here, in parenthesis, that the low nutrition during the post-parturient period is largely responsible for cases of hopeless insanity.) The post-parturient woman's condition cries most loudly for nutrition for healing and repair. A nutritious, non-stimulating diet never harmed anyone, and I do not believe a single physician, when he is sick, practices what some of them preach on that subject. The second class is that of insanity of utero-gestation. This kind of insanity ought to be kept in abeyance until the critical period has passed, but requires careful watching and treatment. Then we have the insanity of lactation, which may usually be averted or arrested in its incipiency by weaning the child and taking it away from the mother and supplying other nutrition, securing private sleep every night, quiet during the day, by means of maltine preparations, and the best possible non-stimulating nutrition.

Then we have general paralysis of the insane. These patients, being never cured, and generally as happy in one place as in another, and seldom dangerous, may be treated as well at home or by traveling as elsewhere, providing they submit without annoyance and danger to themselves or their property, or to others. In cases of acute psychic disturbance, secondary upon temporary hyperæmia, where the patient is conscious something is wrong, and will receive treatment, he may be treated at home. These cases, however, require great vigilance if they have any delusions or suspicions that may lead to homicide or suicide. Delusions and hallucinations based upon auditory diseases render these cases generally unsafe to be treated at home. The advice that is to keep these patients out of the asylum should be cautiously given, and based upon ample means of the friends to keep the patient under constant control. Then we have cases of delirium tremens, mania potu, which may soon be restored by judicious treatment familiar to every physician. Then I proceed to senile dementia, a case of harmless insanity in an old man, a lost mind, which comes from the degenerative changes familiar to us all. No medical man who justly appreciates his position would consign such a patient to the hospital if it be within the power of the family to take care of him. Then insanity connected with far advanced phthisis, unless violent and destructive, is a form not proper to be sent to an asylum. Mild cases of melancholia, where the patient can afford the expense of a medical attendant away from his immediate home, should not be sent to the asylum. These cases are, however, sometimes the most treacherous ones the physician encounters. It is out of them that the daily harvest of suicides is largely made up. These apparently harmless melancholics are the ones whom the physician interrogates with the greatest care. They are not safe either for the physicians or for the friends to be treated at home.

In the new era in medicine now inaugurated by the creation of chairs in psychological medicine in the medical colleges, and the disposition to diffusion of the literature of insanity through the medical profession generally, we shall all soon be quite as well qualified to intelligently advise concerning insanity as concerning other diseases. I have more than once seen patients, who, at home, had given the greatest trouble, when no one at home had the temerity to dare tell him he was insane, cured by telling him candidly that he had been adjudged by a jury to be insane, and was now in an institution provided by the State for the cure of the insane, and that he, although in a different degree from the others around him, was no less insane than those about him.

The important problem in psychiatry, for forensic purposes is that of the differentiation.

[The paper closed with a discussion of the rights of the insane, in which the author maintained the general principle that "no man of unsound mind should be divested of his liberty unless he was dangerous to himself or to others."]—*Cincinnati Lancet and Clinic.*

